# The influence of different light spectra on physiological responses, antioxidant capacity and chemical compositions in two holy basil cultivars

**DOI:** 10.1038/s41598-021-04577-x

**Published:** 2022-01-12

**Authors:** Panita Chutimanukul, Praderm Wanichananan, Supattana Janta, Theerayut Toojinda, Clive Terence Darwell, Kriengkrai Mosaleeyanon

**Affiliations:** National Center for Genetic Engineering and Biotechnology (BIOTEC), National Science and Technology Development Agency, Klong Nueng, Klong Luang, 12120 Thailand

**Keywords:** Biotechnology, Plant sciences

## Abstract

Light-emitting diodes (LEDs) are an artificial light source used in indoor cultivation to influence plant growth, photosynthesis performance and secondary metabolite synthesis. Holy basil plants (*Ocimum tenuiflorum*) were cultivated under fully controlled environmental conditions with different red (R) and blue (B) light intensity ratios (3R:1B, 1R:1B and 1R:3B), along with combined green (G) LED (2R:1G:2B). The photosynthetic activities of both cultivars were maximal under 3R:1B. However, the highest fresh (FW) and dry (DW) weight values of green holy basil were recorded under 3R:1B and 2R:1G:2B, significantly higher than those under alternative light conditions. For red holy basil, the highest FW and DW were recorded under 1R:3B. Moreover, 2R:1G:2B treatment promoted pigment (chlorophyll and carotenoid) accumulation in green holy basil, while red holy basil was found to be rich in both pigments under 3R:1B. Antioxidant capacity was also influenced by light spectrum, resulting in greater total phenolic content (TPC) and DPPH accumulation in both cultivars under 1R:3B. The highest content of flavonoid in green holy basil was detected under 1R:1B; meanwhile, 1R:3B treatment significantly promoted flavonoid content in red holy basil. In addition, anthocyanin content increased in red holy basil under 1R:3B conditions. Gas chromatography coupled with mass spectrometry (GC–MS/MS) analysis of chemical composition showed higher proportional accumulation in Methyleugenol and Caryophyllene of two cultivars grown under all light spectrum ratios at two developmental stages. Overall, specific light spectrum ratios induced different chemical composition responses in each cultivar and at each developmental stage. These results suggest that 3R:1B was favorable for biomass accumulation and photosynthetic responses in green holy basil, while 1R:3B provided antioxidant accumulation. For red holy basil cultivation, 1R:3B provided optimal growing conditions, promoting improvements in plant biomass, and physiological and antioxidant capacities.

## Introduction

Holy basil (*Ocimum tenuiflorum* L.) commonly known as Kaphrao in Thai or Tulsi in Hindi is widely cultivated worldwide^1,2^, including Southeast Asia and especially in Thailand^3^. There are two common cultivars, *green* and *red* leaved: green holy basil has medium green leaves with green stem, while red holy basil has dark green leaves, reddish purple stems with a strong spicy flavor and taste^4,5^. Whilst commonly used as a herbal remedy^6,7^. In holy basil, the chemical components are rich in two major groups of secondary metabolites as phenyl propanoids and terpenoids. Morover, Kelm et al.^8^ and Singh and Chaudhuri^9^ reported methyl eigenol, eugenol, cirsilineol, isothymusin, isothymonin and rosmarinic acid identified from holy basil extracts. Holy basil has shown potential industrial applications in the preparation of pharmaceutical substances and cosmetics^10–12^.

The conditions of plant growth such as light spectrum, temperature, watering, and nutrients play a crucial role in the growth, yield, and secondary compound accumulation in herb plants. Among these, light is a key abiotic factor driving the photosynthetic generation of endogenous chemical energy and regulating physiological signal responses in plants. Primary and secondary responses are triggered by changes in light spectral quality (400–700 nm), intensity, and photoperiod^2,13,14^. Light quality also affects complicated responses in plant physiology, morphology, and gene expression by initiating the signaling of phytochromes, phototropins, and cryptochromes in photoreceptors^15,16^ to regulate plant growth, morphological processes, chloroplast accumulation, and secondary metabolite biosynthesis^17–19^. Red (R) and blue (B) spectrums are well known to promote primary metabolites such as carbohydrates and are strongly absorbed by chlorophyll a and b^20^. Insufficient R and B intensity is shown to reduce photosynthetic rate, leading to lower sugar accumulation and plant growth^21^.

Light-emitting diodes (LEDs) are artificial light sources that offer high energy efficiency combined with low heat emissions. Moreover, LEDs can adjust light intensity and light spectra to promote plant growth and development. There are many studies that have investigated the effects of R and B light quality on growth, photosynthetic activity, and antioxidant capacity, among indoor herbs and vegetables^22,23^. For R light, an emission peak around 660 nm causes notable increases in sugar and starch accumulation in lettuce compared with white light^24^. While lettuce plants grown under only R light showed highly elongated appearance in hypocotyls and cotyledon^25^. B light (approx. 400–500 nm) plays an important role in controlling stomatal opening^26^. Moreover, plants grown under the B light are usually shorter with smaller and thicker leaves. B light can enhance phenolic biosynthesis through the phenylpropanoid pathway in red leaf lettuce, although this has not been shown in sweet basil^23^. Although most studies have shown the important influence of R and B light, there is also evidence that green (G) light (peaking around 510 nm) plays an important role in regulating physiological responses and secondary metabolite biosyntheses such as sugar content in strawberries^27^, total polyphenol content in lettuce^28^, and vitamin C levels in tomatoes and spinach^24,27^. Therefore, it would be of interest to evaluate the influence of G light in improving secondary metabolite syntheses including antioxidant characteristics, phenolic compounds, anthocyanin, and other compounds.

Moreover, secondary metabolites in plants, such as volatile oils, polyphenols, alkaloids, flavonoids, and other compounds, can be influenced by light spectra including the different intensity ratios of R and B light. For example, Carvalho^29^ and Pennisi^30^ reported that the influence of LEDs using R and B light affected photosynthetic activity, antioxidant capacity, and volatile profiles in sweet basil (*Ocimum basillicum*). Secondary compound accumulation also varied according to plant tissue and organs at different stages of plant development^31,32^.

For holy basil, the appropriate ratios of the R and B light spectrum for the cultivation of holy basil remains unclear. The goal of the study is to compare the effects of different light spectra of R and B light ratios from LEDs on physiological responses and biochemical adaptations in two holy basil cultivars. Further, we evaluate the photosynthetic responses in carbon fixation and plant light reaction (the initial photosynthesis stage involving light to chemical energy conversion; ATP and NADPH) under R and B light intensity ratios and evaluate changes in antioxidant capacity and chemical profile in green and red holy basil during the harvesting stage.

## Materials and methods

### Plant material and growth conditions

Seeds of *Ocimum tenuiflorum* from the green and red cultivars were purchased from a commercial seed company (Chia Tai Co. Ltd., Bangkok, Thailand). All seeds were sown and on a 300-cell germination sponge (28.5 × 58 × 3 cm) (ESPEC Corp., Japan), and germinated under the environmental conditions of 150 µmol m^−2^ s^−1^ photosynthetic photon flux density (PPFD) with 16 h d^−1^ photoperiod, 25±1.5 °C air temperature, and 70 ± 5% relative humidity (RH). On day 14 after sowing, well-developed seedlings with true leaves and roots were transplanted into a deep-flow-technique (DFT) hydroponic system in a plant factory with artificial light (PFAL) where plants are cultivated until harvest under fully controlled environmental conditions^33^. The planting density was 22.5 plants m^−2^ by spacing 12 plants in each hydroponic foam (59 × 90 × 3 cm). Three days after transplanting the pH and EC of the modified Enshi nutrient solution^34^ were maintained at 6.5 ± 0.15 and 2 ± 0.02, respectively. Plant growth conditions in PFAL were16 h d^−1^ photoperiod, 25 ± 1 °C, 70 ± 5% RH, and 1000 ± 100 µmol·mol^−1^ CO_2_ (for details of the daily environment under PFAL; Supplementary Table S1).

### Spectral light conditions

**Figure 1:**
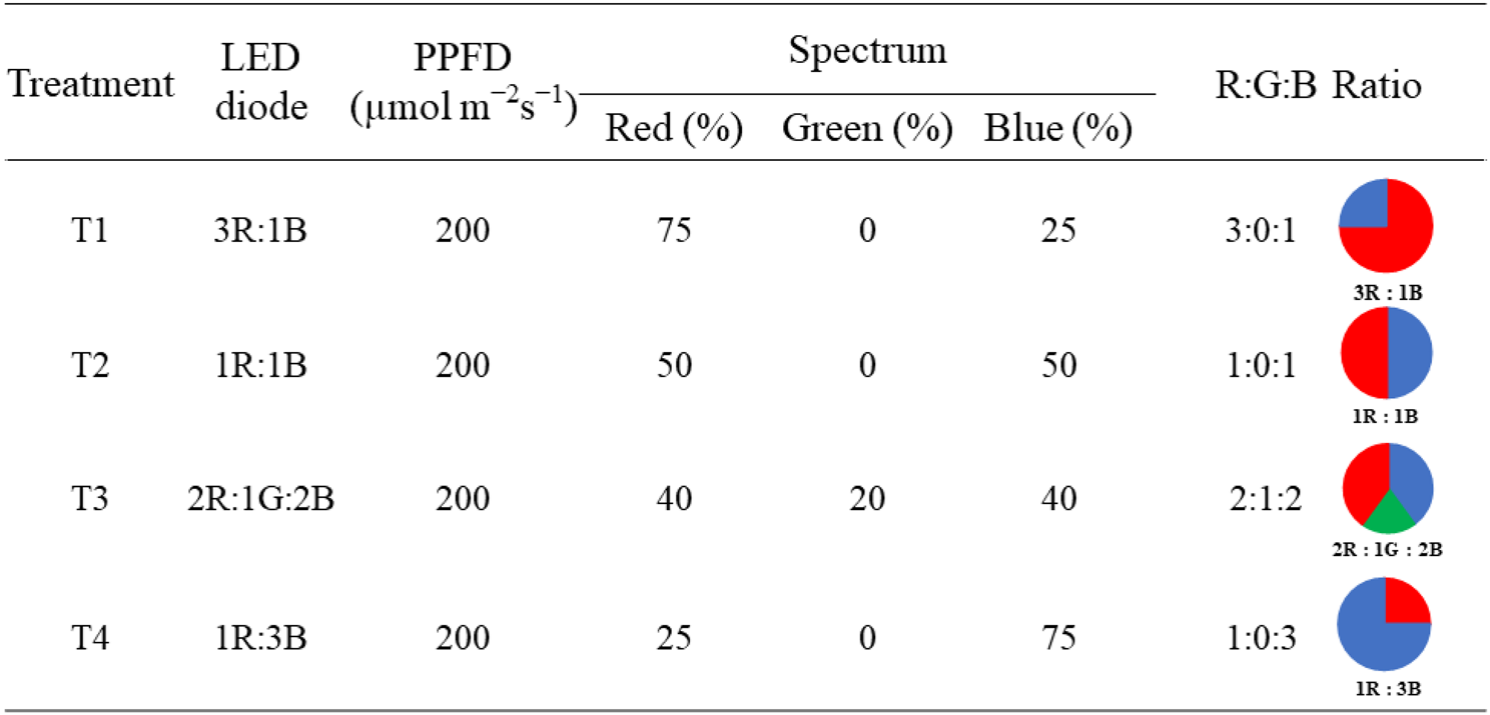
Four different light treatments employed during green and red holy basil growth.

In all experiments, LEDs (AGRI-OPTECH Co., Ltd, Taiwan) elicited R light (λ = 600–700 nm, peak at 660 nm), B light (λ = 400–500 nm, peak at 450 nm), and G light (λ = 500–600 nm, peak at 525 nm). After transplanting, both cultivars were exposed to four different spectral light treatments of R, G, and B ratios including R:G:B = 70:0:25 (T1; 3R:0G:1B), R:G:B = 50:0:50 (T2; 1R:0G:1B), R:G:B = 40:20:40 (T3; 2R:1G:2B) and R:G:B = 25:0:75 (T4; 1R:0G:3B) as shown in Fig. 1. The LEDs were set horizontally, the height of the bench top from the base to LEDs was 40 cm. The photosynthetic photon flux density (PPFD) of R, B, and G was measured by a spectrometer (LI-180, LI-COR Inc., USA). The alternating intervals of R, G, and B LEDs were individually adjusted by built-in switches. The PPFDs of R, G, and B were measured at the base level of the bench top and were recorded at 200 µmol·m^−2^·s^−1^ for all treatments. The relative spectra of the light treatments were recorded from a light meter (Sekonic C-7000, Japan) (see Supplementary Document S1).

### Physiological measurements

To examine the physiological responses of treated plants at different light spectra, the following characteristics were evaluated on day 35 (vegetative stage) and day 42 (flowering stage) after transplantation (DAT). Gas exchange was measured by a portable photosynthesis system (LI-6800, LICOR Inc., Lincoln, NE) connected to a standard 2 cm^2^ chamber. Gas exchange parameters were: net photosynthetic rate (*Pn*), stomatal conductance (*gs*), internal concentration (*Ci*), and transpiration (*E*). Chlorophyll fluorescence parameters including PhiPS2 and ETR were determined. Water use efficiency (WUE) was calculated by dividing *Pn* by *E*, and NPQ was calculated from (Fm/Fm′)-1^38^. Measurement of gas exchange and chlorophyll fluorescence parameters were performed on the third or fourth fully expanded leaf from the plant apex at vegetative and flowering stages. The middle portions of the uppermost fully expanded leaves were evaluated using the following conditions: the molar flow of air per unit leaf area: 500 mmol·m^−2^·s^−1^; leaf surface photosynthetically active radiation: 200 µmol·m^−2^·s^−1^, leaf temperature: 25°C; and CO_2_ concentration: 1000 µmol·mol^−1^. Light spectrum ratios were applied with the same ratios as used during the treatment. Reflectance spectra parameters between the ranges of 380–790 nm were measured by non-invasive spectro-reflectometers, PolyPen RP 400 (UV-VIS and NIR) (Photon Systems Instruments, Brno, Czech Republic). For measuring reflectance spectra, five holy basil leaves that were fully expanded on the third or fourth node from the top were placed individually on a clip of PolyPen’s measuring head (PolyPen RP 400, Photon Systems Instruments, Prague, Czech Republic). The following reflectance indices from the measurement were calculated: normalized difference vegetation index (NDVI; (RNIR − RRED)/(RNIR + RRED))^35^, greenness index (GI; R554/R677)^36^, carotenoid reflectance indexes (CRI2; (1/R510) − (1/R550))^37^, and water index (WI; R900/R970)^38,39^. The fresh weight (FW) and dry weights (DW) of the above-ground shoots were collected on 35 and 42 DAT of each treatment.

### Biochemical evaluations

#### Sample extraction

After harvest at the vegetative and flowering stages whole plant (leaf, stem, and inflorescence) was dried under hot air at 40 °C for 72 h. Plant extractions were performed on dried tissue using modified methods of Bao et al.^40^. 10 mg of dried samples of holy basil was ground in a mortar to form a fine powder the prepared powder in each sample was extracted with 5 mL of methanol containing 1% HCl and completely mixed in a vortex before being allowed to stand at room temperature for 3 h, or until the powder was pale. Then the mixture was centrifuged at 8000 rpm for 10 min. The supernatant was collected into a new tube (10 mL) and used to determine total phenolic compound, flavonoid content and DPPH radical scavenging.

#### Total phenolic compounds (TPC) and flavonoid content

TPC was determined by modified Folin-Ciocalteu colorimetric methods^40^. 200 µL of the extracts was oxidized with 200 µL of 1 N Folin-Ciocalteu reagent. Sodium carbonate (7.5%) was then added to neutralize the reaction. After incubation for 1 h at 25 °C, the reaction turned blue. The absorbance of the resulting mix was measured at 730 nm with a spectrophotometer (Multiskan Sky, Thermo Scientific). TPC was calculated using the standard gallic acid curve and expressed as milligrams of gallic acid equivalent (mg of GAE) per gram of DW. A colorimetric method^40^ with minor modification was assayed to quantify flavonoid content, 350 µL of the prepared extracts was pipetted into a 2-mL tube, and 75 µL of 5% sodium nitrite (NaNO_2_) was added. The mixture was kept at 25 °C. After 5 min, 75 µL of 10% aluminum chloride (AlCl_3_·6H_2_O) was added and then left to stand for 5 min. 500 µL of 1 M sodium hydroxide (NaOH) was finally added. The mixture solution was well mixed and left at 25 °C for 15 min. The absorbance of the solution was determined at 515 nm by a spectrophotometer. Flavonoid content was measured based on a rutin standard curve, and the result was shown as milligrams of rutin equivalents per gram of DW.

#### DPPH radical scavenging assay

The free radical scavenging activity of holy basil was estimated by the modified method of Bao^40^. 100 µL of the extract was added to 900 µL of 40 µM DPPH. The reaction solution was completely mixed and kept at 25 °C for 15 min. 0.5 mM of Trolox was used as a reference antioxidant. The absorbance of the solution was measured at 515 nm. Antioxidant activity was expressed with inhibition percentage of DPPH absorbance as follows: ((A_control_ − A_515_)/A_control_) × 100^41,42^.

#### Anthocyanin content

The anthocyanin content was measured by spectrophotometer as described by Liang^43^ with minor modifications. The anthocyanin was extracted by incubating 50 mg of powder with 600 µL of extraction solution (1% HCL in methanol) and keeping at 25 °C for 3 h in darkness. Next, 300 µL of deionized water and 300 µ of chloroform were added. The mixture was mixed well and centrifuged at 10,000 rpm for 5 min to remove the suspended substance. The supernatant solution was measured using a spectrophotometer. The absorbance of the reaction mixture was measured at 530 and 675 nm and the anthocyanin content was calculated as the following equation: (A_530_ − 0.33 × A_657_) and presented in µg per gram of DW.

#### Chemical profiles

To investigate the chemical profile of green and red holy basils grown under four different light spectrum ratios, one gram of dry powder was used for extraction in 10 mL of 99.9% methanol and then uniformly mixed by a vortex for 1 min. The extracts were sonicated by ultrasound at 40–45 °C for 30 min. The prepared extracts were centrifuged at 10,000 rpm for 5 min, filtered through 11 µm of Whatman filter paper No.1 (Whatman Grade 1 filter paper) into neat vials, and stored at 4 °C. The prepared solution was used for analyzing the chemical composition by gas chromatography coupled with mass spectrometry (GC–MS/MS).

The analysis of the chemical composition of holy basil in each treatment was analyzed by gas chromatography coupled with mass spectrometry (GC–MS/MS, GCMS TQ8050) (Shimadzu, Japan) equipped with a fused DB-5MS capillary column (dimensions: 30 m × 0.25 mm; × 0.25 µm). The temperature in the oven was increased from 40 to 220 °C with nitrogen gas at a flow rate of 1 mL/min as the carrier. The injection volume of the sample was diluted in 99.9% methanol at the volume ratio of 1:1. The sample was injected with the split mode (split ratio 1:50) with injector temperature at 230 °C and the injection volume 0.1 μL in methanol. The Gas chromatography–mass Spectrometry (GC–MS) analysis of the chemical compounds was carried to Mass Spectrometer (Thermo Scientific ITQ 1100) with TG-5 fused silica capillary column (30 m × 0.32 mm, film thickness 0.32 μm) using helium gas at 1.0 mL/min. MS operated in EI mode at 70 eV, and a scan range of 40–450 amu^44–46^. For each light treatment of green or red holy basil, GC–MS/MS analysis was conducted on four plants using the dry whole plant as a sample. The chemical components data were analyzed and identified by an MS library search in the database of NIST 08 (Thermo Fisher Scientific) and 11^th^ Edition of WILEY MS (Thermo Fisher Scientific Austria), and by comparing with the MS literature data^47^ and also by co-injection of available authenticated samples purchased from Sigma-Aldrich, India (> 98% purity). The relative amounts of individual components were calculated based on the GC peak area (FID response) without using a correction factor. The chemical compositions were calculated from the GC relative as peak areas (%).

### Statistical and data analyses

The physiological analyses experiment, i.e., TPC, antioxidant capacity, and anthocyanin content were designed according to a randomized complete block design (RCBD) with four replications (three plants of each cultivar per replication). Statistical analysis was conducted with IBM SPSS (IBM Corporation; Armonk, NY, USA). One-way analysis of variance (ANOVA) was used to evaluate the data for each parameter. The mean ± SE (standard error) were analyzed with Duncan’s multiple range test (DMRT) using significance tested at the p < 0.05 level.

In order to investigate the relationships between different variables in two cultivars and two developmental stages: biomass accumulation, physiological response parameters and antioxidant capacity and anthocyanin content depending on the four different light spectrum ratios (T1; 3R:1B, T2; 1R:1B, T3; 2R:1G:2B and T4; 1R:3B). A principal component analysis (PCA) was conducted using the SAS statistical software package (Cary, NC). Further, the relationship between traits (physiological response, antioxidant capacity and anthocyanin content) were assessed using Pearson’s correlation coefficient test. Additionally, a hierarchical clustering analysis was performed using Ward’s method (also in SAS) which outputs results in heat map format to facilitate a visual assessment of clustering and key determinants.

### Research involving plants

The present study complied with the international and national guidelines for the use of experimental plants.

## Result

### Biomass accumulation and plant height

**Figure 2:**
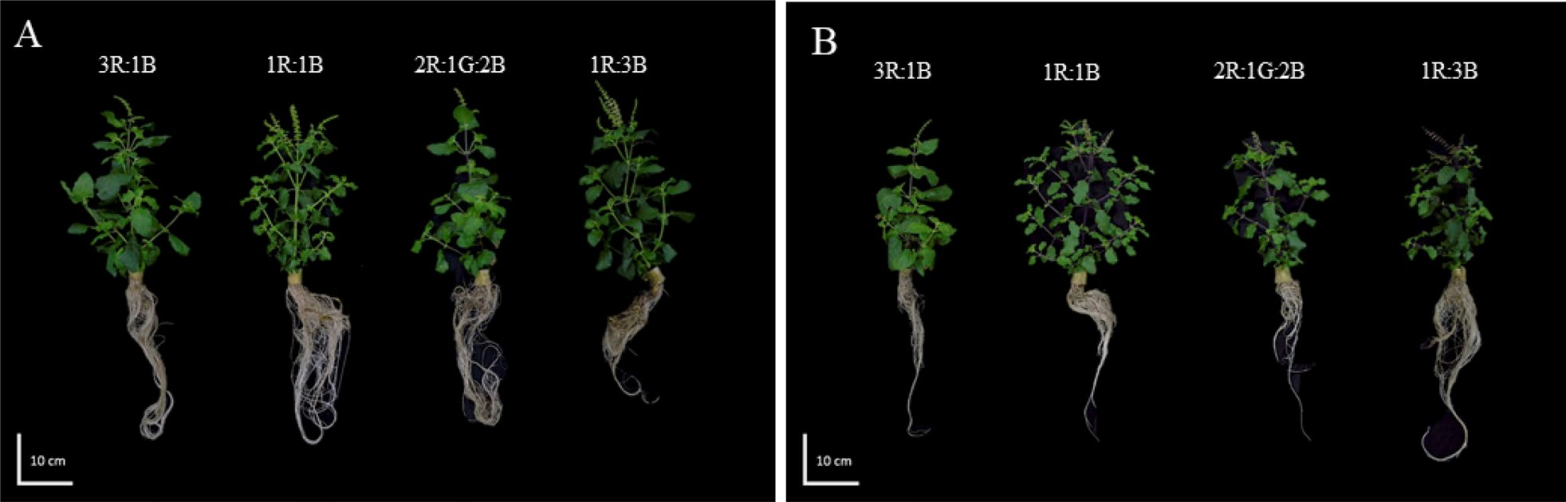
The phenotypes of green holy basil (**A**) and red holy basil (**B**) under four light treatments (3R:1B, 1R:1B, 2R:1G:2B, and 1R:3B) at flowering stage. R, G and B represent the red, green and blue llight spectra, respectively, and number before the letters stand for the ratio of each lighrt spectra.

The effects of different light treatments on two cultivars of green and red holy basil during the vegetative and flowering stages were evaluated. The results of plant growth and height are shown in Table 1. Growth phenotypes at the flowering stage are shown in Fig. 2 (see Fig. S1 in Supplementary Document S2 for vegetative stage). At the vegetative and flowering stages for green holy basil, above ground FW and DW were lowest under high blue light ratio (1R:3B). A similar trend was found in plant height at both development stages. Supplemental green light (2R:1G:2B) also resulted in increased FW and shoot elongation when compared with 50:50 red-blue light (1R:1B). Compared with high red light ratio (3R:1B), FW of above ground tissue was significantly increased at the vegetative and flowering stage by approximately 48.81% and 67.27%, respectively. On the other hand, FW and DW of red holy basil at the flowering stage had the highest values in 1R:3B, and was not significantly different in the vegetative stage. The light treatments did not significantly affect plant height of red basil at the developmental stage. These data suggest that different light spectrum treatments cause differential responses for biomass and plant height in each cultivar.

**Table 1:** Biomass and plant height of green and red holy basil at vegetative stage (35 DAT) and flowering stage (42 DAT) under different light spectrum ratios (3R:1B, 1R:1B, 2R:1G:2B, and 1R:3B).

Cultivar	Light treatment	Fresh weight (g)		Dry weight (g)		Height (cm)	
		Vegetative	Flowering	Vegetative	Flowering	Vegetative	Flowering
Green	3R:1B	12.59 ± 0.30a	36.08 ± 2.072a	1.65 ± 0.07	4.23 ± 0.15a	19.5 ± 0.93a	33.21 ± 2.56a
	1R:1B	13.89 ± 1.56a	25.19 ± 1.96b	1.86 ± 0.29	2.96 ± 0.63bc	21.17 ± 1.34a	27.04 ± 2.65ab
	2R:1G:2B	11.19 ± 1.23ab	39.89 ± 3.68a	1.41 ± 0.13	3.94 ± 0.34a	19.08 ± 0.85a	32.25 ± 2.66a
	1R:3B	8.46 ± 0.41b	21.57 ± 1.98b	1.18 ± 0.13	2.65 ± 0.23c	13.79 ± 1.07b	22.54 ± 1.71b
Red	3R:1B	6.16 ± 0.73	15.01 ± 0.73b	0.82 ± 0.08	1.95 ± 0.07b	15.96 ± 1.26	22.13 ± 0.72
	1R:1B	5.96 ± 0.16	14.17 ± 0.46bc	0.78 ± 0.05	1.76 ± 0.04b	14.58 ± 0.29	21.13 ± 1.26
	2R:1G:2B	6.56 ± 0.51	15.96 ± 0.17b	0.83 ± 0.06	1.85 ± 0.03b	17.13 ± 0.74	23.29 ± 1.53
	1R:3B	6.08 ± 0.50	17.43 ± 0.14a	0.85 ± 0.05	2.31 ± 0.16a	14.17 ± 0.73	20.29 ± 1.01

R, G and B represent the red, green and blue llight spectra, respectively, and number before the letters stand for the ratio of each lighrt spectra**. **Lower-case letters after the same parameter indicate significant differences (p < 0.05). Values are represented as mean ± SE (n = 4). ANOVA was performed followed by mean comparison with DMRT.

### Physiological responses

**Figure 3:**
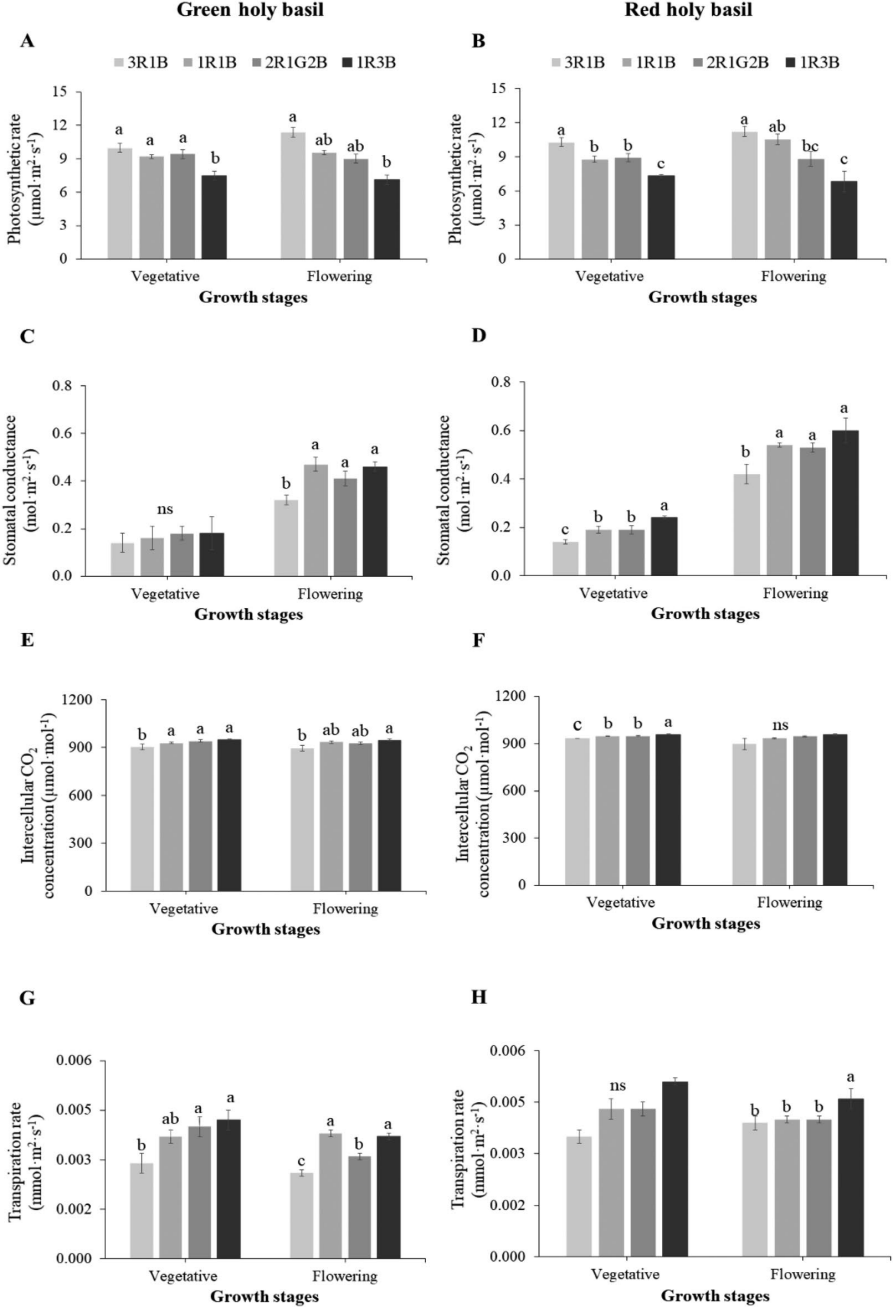
Effects of different light spectrum treatments on net photosynthesis rate (*Pn*, **A**,**B**), stomatal conductance (*g*_*s*_, **C**,**D**), intercellular CO_2_ concentration (*Ci*, **E**,**F**), and transpiration rate (*E*, **G**,**H**) of green and red holy basil at vegetative and flowering stages. R, G and B represent the red, green and blue llight spectra, respectively, and number before the letters stand for the ratio of each lighrt spectra. Values are represented as mean ± SE (n = 4). ANOVA was performed followed by mean comparison with DMRT. Lower-case letters above bars indicate significant difference (p < 0.05); “ns” indicates no significant difference. Bars indicate standard errors.

The results of the photosynthetic performance study showed that *Pn* values of both cultivars varied according to light spectrum regime. *Pn* of green and red holy basils showed the highest values under 3R:1B lighting during both vegetative and flowering stages. 1R:3B caused a decrease in *Pn* for both holy basil cultivars (Fig. 3A,B). 1R:1B and 2R:1G:2B showed the same values of *Pn* in 3R:1B in green holy basil (Fig. 3A). However, *Pn* of red basil under both 1R:1B and 2R:1G:2B conditions was the same, and had a slightly decreased *Pn* when compared with 3R:1B at both vegetative and flowering stages (Fig. 3B). During the growth development stage, green holy basil was not significantly different in *gs* under the four light treatments (Fig. 3C). Red holy basil was clearly affected by light spectra. A decrease in *gs* was identified at both development stages under 3R:1B and was highest under 1R:3B, which concurred with the finding of photosynthetic responses at vegetative and flowering stage (Fig. 3D,E). At vegetative stage, the red holy basil under 3R:1B also had significantly lower *Ci* than that under 1R:3B treatment, while *Ci* was not significantly different at flowering stage (Fig. 3F). *E* of both cultivars was consistent with their *gs* in each light spectrum and showed the highest value under 1R:3B, whereas the change in *E* was opposite to that observed for *Ci* concentration (Fig. 3G,H).

**Figure 4:**
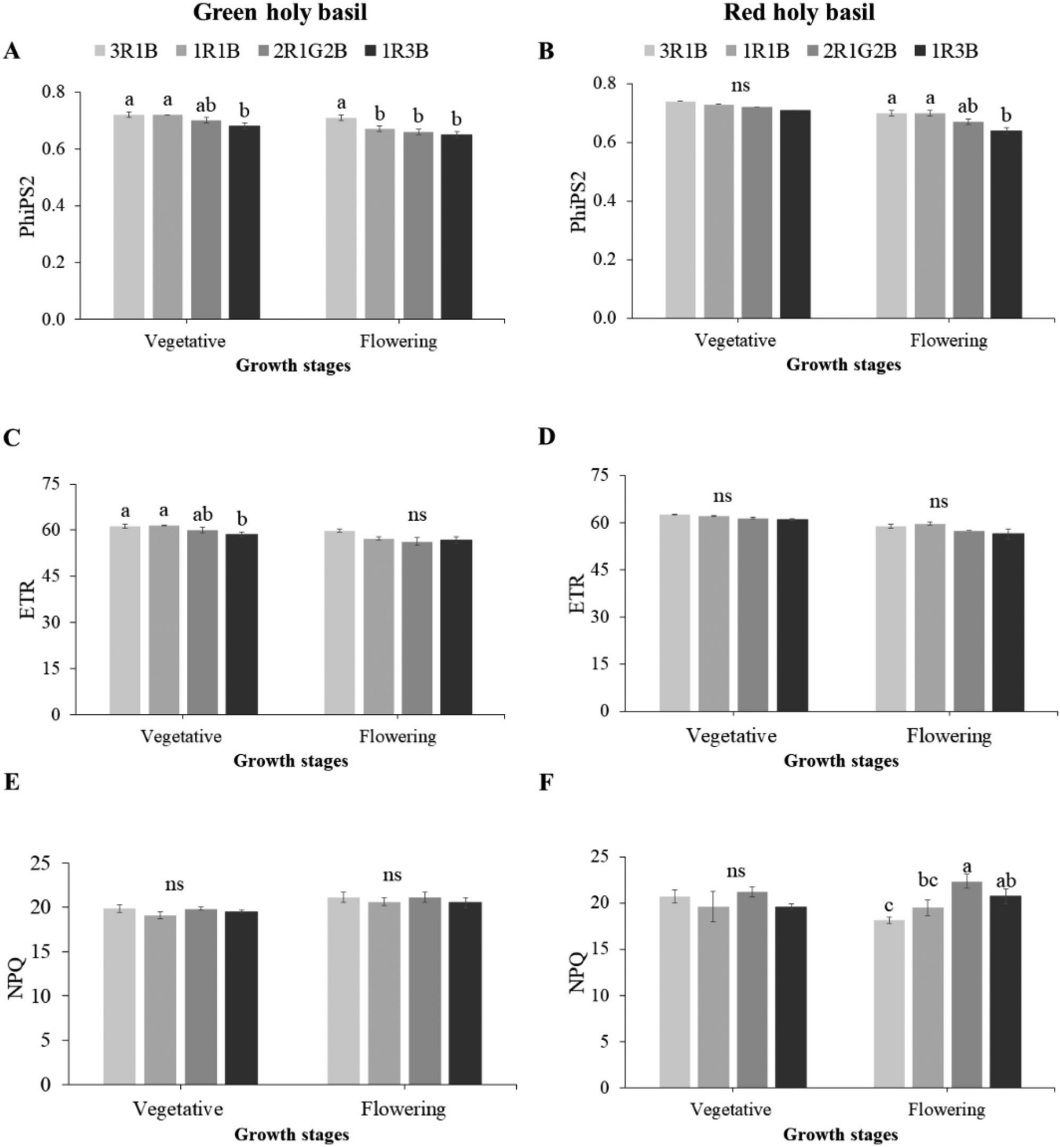
Photosystem II efficiency (PhiPS2, **A**,**B**), electron transport rate (ETR, **C**,**D**), and non-photochemical quenching (NPQ, **E**,**F**) of green and red holy basil at vegetative and flowering stages under four different light treatments (3R:1B, 1R:1B, 2R:1G:2B, and 1R:3B). R, G and B represent the red, green and blue llight spectra, respectively, and number before the letters stand for the ratio of each lighrt spectra. Values are represented as mean ± SE (n = 4). ANOVA was performed followed by mean comparison with DMRT. Lower-case letters above bars indicate significant difference (p < 0.05); “ns” indicates no significant difference. Bars indicate standard errors.

In order to investigate light spectrum ratio effects on light response system activity, PhiPS2 (Photosystem II efficiency under a light condition), electron transport rate (ETR), and non-photochemical quenching (NPQ) were examined. Light treatments significantly affected PhiPS2 of green holy basil under both development stages (Fig. 4A). However, light treatment significantly affected PhiPS2 of red holy basil during flowering stage only (Fig. 4B). Additionally, ETR during flowering stage was significantly different in green holy basil. The ratio of 3R:1B and 1R:1B recorded the highest, while 1R:3B showed the lowest value. However, red holy basil did not show significant differences at either development stage (Fig. 4C,D). Overall, the NPQ of green holy basil under different light treatment was not significantly different at either vegetative or reproductive stages (Fig. 4E). Red holy basil showed significant differences in NPQ during flowering stage (Fig. 4F). Moreover, 3R:1B in flowering stage had the lowest NPQ, while supplemental green light (2R:1G:2B) recorded less than the highest value.

**Figure 5:**
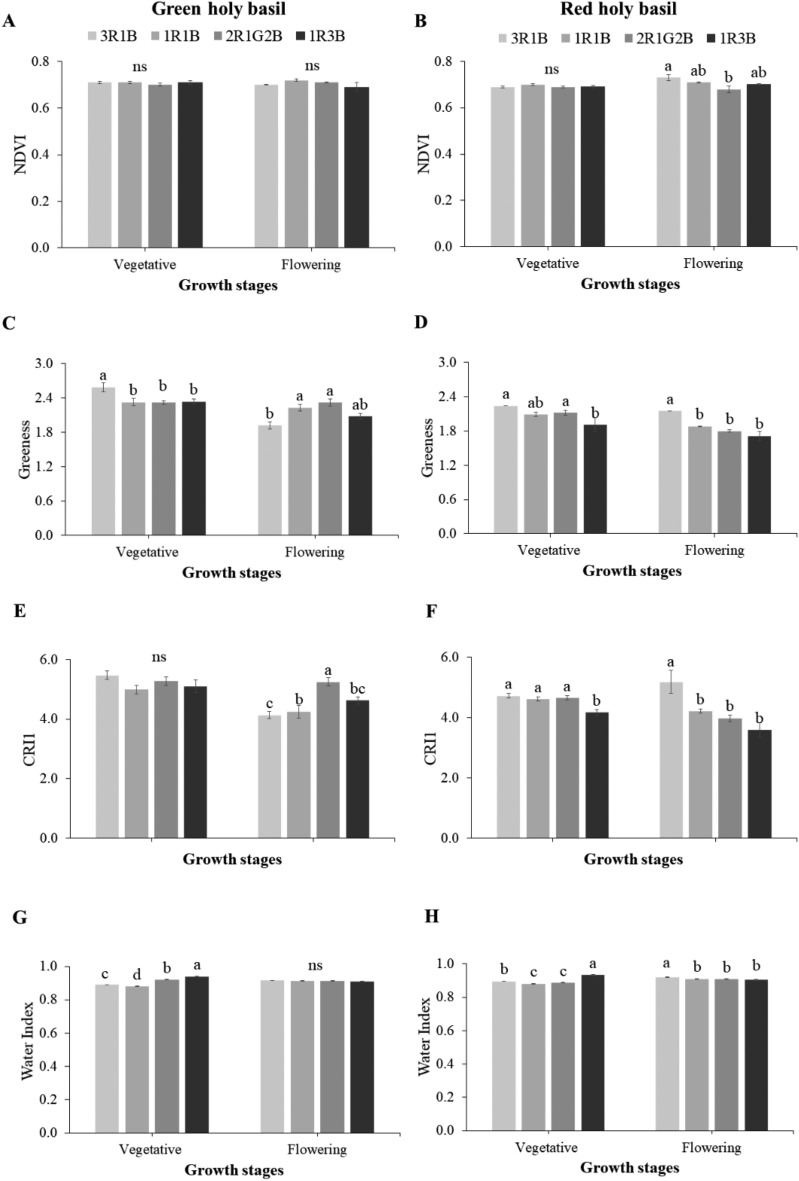
Reflectance spectral indices, normalized difference vegetation index (NDVI), greenness index (G), carotenoid reflectance indexes (CRI2) and water index (WI) of green and red holy basil at vegetative and flowering stages under four different light treatments (3R:1B, 1R:1B, 2R:1G:2B, and 1R:3B). R, G and B represent the red, green and blue llight spectra, respectively, and number before the letters stand for the ratio of each lighrt spectra. Values are represented as mean ± SE (n = 4). ANOVA was performed followed by mean comparison with DMRT. Lower-case letters above bars indicate significant difference (p < 0.05); “ns” indicates no significant difference. Bars indicate standard errors.

 The result of reflectance spectrum indices in each treatment is shown in Fig. 5. Different light spectra did not affect NDVI at the seedling and flowering stage in green holy basil. However, light spectra treatment significantly affected NDVI at the flowering stage of red holy basil (Fig. 5A,B). The high red light ratio (3R:1B) showed the highest value whilst green light supplemented (2R:1G:2B) revealed the lowest (Fig. 5B). Leaf chlorophyll level and carotenoid pigments were also investigated using GI and CRI2 indices, respectively. After both holy basil cultivars were exposed to difference light spectrum ratios at the vegetative and reproductive stage, significant differences in GI were detected. For green holy basil, 3R:1B had the highest index in the vegetative stage but the lowest index value in flowering stage (Fig 5C). Red holy basil showed a significant difference between light spectrum ratios. GI of red holy basil in both development stage was the highest under high red light (3R:1B) conditions, while high blue light conditions (1R:3B) recorded the lowest (Fig. 5D). The similar results were also detected in the CRI1 index at the vegetative and flowering stages of both cultivars (Fig. 5E,F). For plant water status, WI of green and red holy basil under 1R:3B treatment was significantly higher in comparison with other light treatments at the vegetative stage (Fig. 5G,H). For red holy basil, there were significant differences between light spectra ratios at the flowering stage, WI was highest in plants grown under 3R:1B (Fig. 5H).

### Antioxidant properties and anthocyanin content

**Figure 6:**
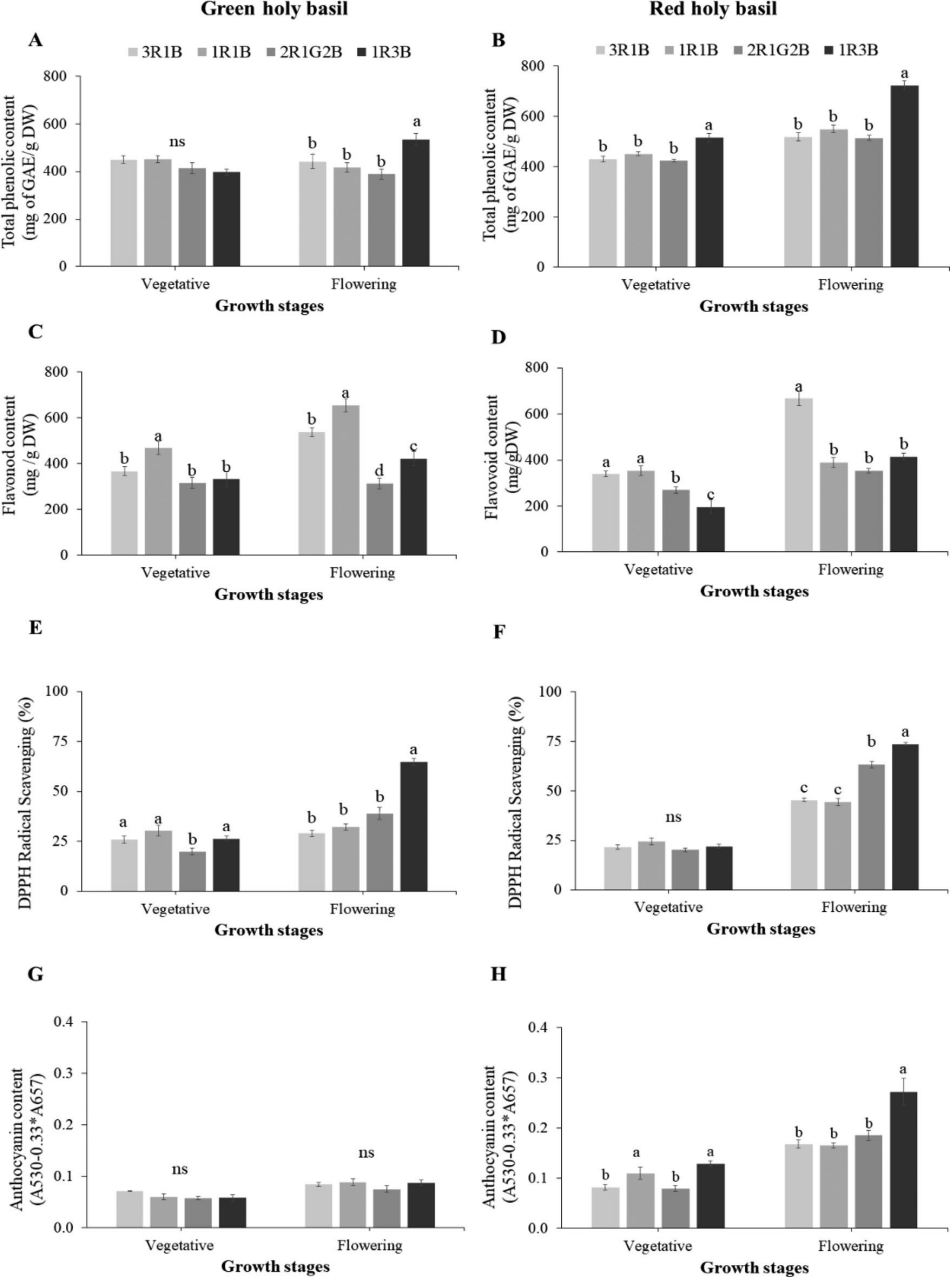
The content of total phenolic content (TPC), flavonoid content, DPPH radical scavenging, and anthocyanin content of green and red holy basil at vegetative and flowering stages under four different light treatments **(**3R:1B, 1R:1B, 2R:1G:2B, and 1R:3B**)**. R, G and B represent the red, green and blue llight spectra, respectively, and number before the letters stand for the ratio of each lighrt spectra. Values are represented as mean ± SE (n = 4). ANOVA was performed followed by mean comparison with DMRT. Lower-case letters above bars indicate significant differences (p < 0.05); “ns” indicates no significant difference. Bars indicate standard errors.

The contents of TPC, flavonoids, DPPH and anthocyanin were affected by different light spectrum ratios (Fig. 6). The TPC content in each cultivar was notably highest in 1R:3B treatment (Fig. 6A,B). The flavonoids content of vegetative and flowering reached maximum at 1R:1B treatment stage in green holy basil (Fig. 6C). Whereas, flavonoid content of both development stages was increased in 3R:1B treatment in red holy basil (Fig. 6D). DPPH radical scavenging activity showed a similar result as TPC for both green and red holy basils, and DPPH was significantly highest in 1R:3B treatment (Fig. 6E,F). However, the supplementary green-light induced greater DPPH than the 3R:1B and 1R:1B treatments for both green and red holy basils at the flowering stage (Fig. 6E,F).

In green holy basil, there were no significant differences in anthocyanin content under different light spectrum ratios at the vegetative stage and flowering stage (Fig. 6G). On the other hand, different light spectrum ratios affected anthocyanin accumulation in red basil. Anthocyanin content increased with high B light ratio (1R:3B) when compared with other spectrum ratios (Fig. 6H).

### Principal component analysis and correlation analysis

**Figure 7:**
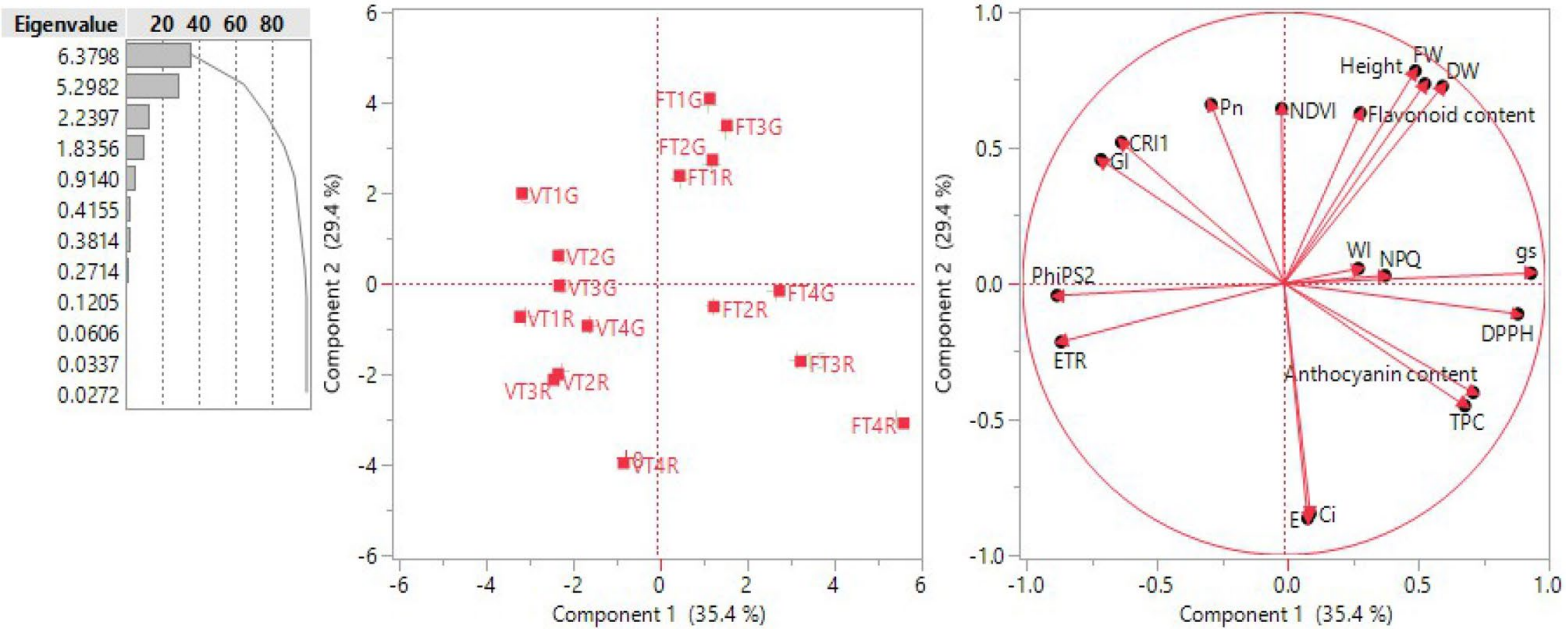
Principal components analysis using the correlation matrix built using data for differential biomass accumulation, physiological response parameters and antioxidant capacity in the two cultivars and two developmental stages grown under four different light spectrum ratios (T1; 3R:1B, T2; 1R:1B, T3; 2R:1G:2B and T4; 1R:3B). R, G and B represent the red, green and blue llight spectra, respectively, and number before the letters stand for the ratio of each lighrt spectra.

Principal component analysis (PCA) was conducted in order to investigate the relationships between all measured variables in (Fig. 7). The PCA indicates a division according to development stage with further structure resulting from lighting treatment. The first principal component (explaining 35.4% of the variance) relates to variation in TPC, DPPH and anthocyanin content, *gsw*, NPQ and WI at flowering stage of red holy basil under 1R:1B, 2R:1G:2B, 1R:1B and 1R:3B treatments, and to those at flowering stage of green holy basil under 1R:3B. The second principal component (29.4%) relates to values of FW, DW, height, flavonoid content and NDVI as well as the values of *Ci* and *E* associated with red holy basil under 1R:3B at vegetative stage. Correlations among traits is shown in Supplementary Table S2. The strongest correlation is between PhiPSII and ETR (*r* = 0.9756, *P* < 0.001), FW and DW (*r* = 0.9591, *P* < 0.001), and CRI and GI (*r* = 0.9545, *P* < 0.001). Among photosynthetic traits, *Pn* displayed strong negative correlation with *Ci* (*r* = -0.8357, *P* < 0.001) and *gs* was negatively correlated with PhiPSII (*r* = 0.776, *P* < 0.001) and ETR (*r* = 0.7901, *P* < 0.001). *E* was also positively correlated with *Ci* (*r* = 0.7843, *P* < 0.001).

**Figure 8:**
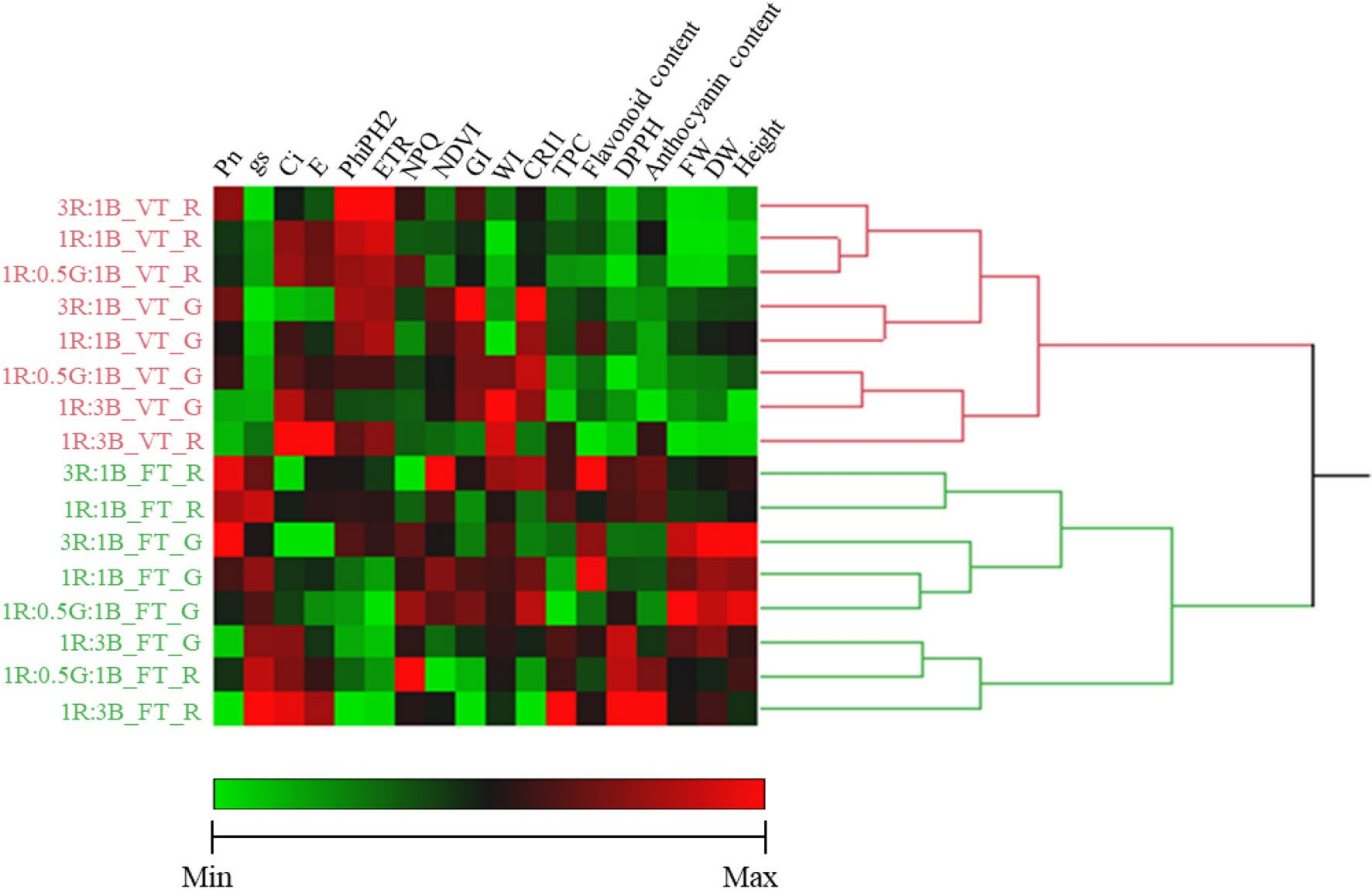
Heat map and clustering analysis of differential biomass accumulation, physiological response parameters and antioxidant capacity among different light treatments (3R:1B, 1R:1B, 2R:1G:2B, 1R:3B) in green (G) and red (R) holy basil at vegetative (VT) and flowering (FT) stages. R, G and B represent the red, green and blue llight spectra, respectively, and number before the letters stand for the ratio of each lighrt spectra.

JMP clustering to evaluate treatment effects indicates that cultivar developmental stage separates empirical measures into two distinct clusters (Fig. 8). Within these development stage clusters, cultivar variety is often split. However, light ratio appears significant as some treatments are regularly grouped. For example, 3R:1B and 1R:1B are regularly paired while 1R:3B treatments are typically clustered alongside 2R:1G:2B.

### Chemical profile

In holy basil leaves, four chemical classes were found: monoterpenes, phenylpropanoids, sesquiterpenes, and diterpenes. Green holy basil at vegetative stage showed higher proportions of methyl eugenol in all light treatments having approximately 83.94%, 87.52%, 85.53% and 79.59% under 3R:1B, 1R:1B, 2R:1G:2B, and 1R:3B treatments, respectively (Table 2). Caryophyllene was also present in considerable proportions. All light treatments were found to be rich in total phenylpropanoids, while total monoterpenoids and total diteroenoids could not be detected under 2R:1G:2B and 1R:1B, respectively. Moreover, only 3R:1B induced β-elemene (0.48%). Similar patterns of chemical profile (for methyl eugenol and caryophyllene) were also observed in green holy basil at flowering stage under all light treatments (Table 3). However, under 1R:3B treatment green holy basil showed a large proportion of methyl eugenol (80.85%). Total sesquiterpenoid was induced when the plant was exposed to 3R:1B (24.72%) and 2R:1G:2B (22.85%). Moreover, green holy basil grown under 3R:1B conditions produced more sesquiterpene compounds than under other light conditions. Monoterpenoids were present in very low quantities under all lights treatments, while diterpenoids were suppressed under the 3R:1B condition.

**Table 2:** Chemical profiling of green holy basil under different light spectrum ratios (3R:1B, 1R:1B, 2R:1G:2B, and 1R:3B) at vegetative stage.

Compound	Molecular formula	Class	RT (min)	Peak area %			
				3R:1B	1R:1B	2R:1G:2B	1R:3B
Eugenol	C_10_H_12_O_2_	Phenylpropanoid	21.613	1.33	nd	nd	1.39
Endo-Borneol	C_10_H_18_O	Monoterpenoid	13.814	nd	0.33	nd	0.40
Citronellol	C_10_H_20_O	Monoterpenoid	41.846	0.35	nd	nd	nd
Methyleugenol	C_11_H_14_O_2_	Phenylpropanoid	23.786	82.61	87.52	85.53	78.19
Caryophyllene	C_15_H_24_	Sesquiterpenoid	24.453	12.29	7.77	10.26	13.11
Humulene	C_15_H_24_	Sesquiterpenoid	25.929	0.52	0.27	0.52	0.67
α-Guaiene	C_15_H_24_	Sesquiterpenoid	28.053	1.45	nd	0.08	1.49
α-Copaene	C_15_H_24_	Sesquiterpenoid	22.619	nd	0.98	0.34	0.96
Germacrene D	C_15_H_24_	Sesquiterpenoid	26.984	nd	2.22	1.59	2.21
β-elemene	C_15_H_24_	Sesquiterpenoid	23.212	0.48	nd	nd	nd
Caryophyllene oxide	C_15_H_24_O	Sesquiterpenoid	30.977	nd	0.91	0.98	1.13
Neophytadiene	C_20_H_38_	Diterenoid	40.399	0.96	nd	0.69	0.45
Total phenylpropanoid				83.94	87.52	85.53	79.59
Total sesquiterpenoid				14.75	12.15	13.78	19.56
Total monoterpenoid				0.35	0.33	0.00	0.40
Total diterpenoid				0.96	0.00	0.69	0.45
Total				100.00	100.00	100.00	100.00

“nd” indicates not detected. R, G and B represent the red, green and blue llight spectra, respectively, and number before the letters stand for the ratio of each lighrt spectra.

**Table 3:** Chemical profiling of green holy basil under different light spectrum ratios (3R:1B, 1R:1B, 2R:1G:2B, and 1R:3B) at flowering stage.

Compound	Molecular formula	Class	RT(min)	Peak area %			
				3R:1B	1R:1B	2R:1G:2B	1R:3B
Eugenol	C_10_H_12_O_2_	Phenylpropanoid	21.613	3.80	nd	nd	nd
Endo-Borneol	C_10_H_18_O	Monoterpenoid	13.814	0.58	0.54	0.54	0.34
Citronellol	C_10_H_20_O	Monoterpenoid	41.846	0.24	0.36	nd	nd
Methyleugenol	C_11_H_14_O_2_	Phenylpropanoid	23.786	70.38	78.29	75.83	80.85
Caryophyllene	C_15_H_24_	Sesquiterpenoid	24.453	12.94	9.82	14.94	10.24
Humulene	C_15_H_24_	Sesquiterpenoid	25.929	0.55	0.29	0.61	0.33
α-Guaiene	C_15_H_24_	Sesquiterpenoid	28.053	3.57	3.11	2.28	3.28
Germacrene B	C_15_H_24_	Sesquiterpenoid	28.052	nd	nd	nd	0.64
Germacrene D	C_15_H_24_	Sesquiterpenoid	26.984	4.83	4.16	3.94	2.12
Caryophyllene oxide	C_15_H_24_O	Sesquiterpenoid	30.977	0.88	nd	nd	nd
Azulene	C_15_H_24_	Sesquiterpenoid	23.208	0.67	nd	nd	nd
Cadina	C_15_H_24_	Sesquiterpenoid	28.486	0.30	nd	nd	nd
β-Famesene	C_15_H_24_	Sesquiterpenoid	30.968	nd	nd	0.31	nd
Bicyclosesquiphellandrene	C_15_H_24_	Sesquiterpenoid	23.140	0.98	1.20	0.77	0.49
Isodaucene	C_15_H_24_	Sesquiterpenoid	23.245	nd	0.84	nd	nd
Isoledene	C_15_H_24_	Sesquiterpenoid	28.511	nd	0.35	nd	nd
Neophytadiene	C_20_H_38_	Diterenoid	40.399	nd	0.47	nd	0.90
Phytol	C_20_H_40_O	Diterenoid	49.135	0.29	0.58	0.78	0.81
Total phenylpropanoid				74.18	78.29	75.83	80.85
Total sesquiterpenoid				24.72	19.77	22.85	17.10
Total monoterpenoid				0.82	0.90	0.54	0.34
Total diterpenoid				0.29	1.04	0.78	1.70
Total				100.00	100.00	100.00	100.00

“nd” indicates not detected**.** R, G and B represent the red, green and blue llight spectra, respectively, and number before the letters stand for the ratio of each lighrt spectra.

Red holy basil chemical profile at the vegetative stage was mainly composed of methyl eugenol (phenylpropene compound; range 78–81%) under all light treatments (Table 4). β-Acoradiene was identified only under 3R:1B conditions, while Acoradiene was detected for 1R:3B. When grown under 3R:1B and 2R:1G:2B four major chemical compounds were induced: phenylpropanoid, sesquiterpenes, monoterpenoid and diteroenoids. During flowering stage of red holy basil, the dominant identified compounds found was methyl eugenol under all light treatments (Table 5). Additionally, α-bisabolene was only produced under 1R:1B while caryophyllene oxide was suppressed in this condition. Green light supplement (2R:1G:2B) affected the compound composition by suppressing monoterpenoids production.

**Table 4:** Chemical profiling of red holy basil under different light spectrum ratios (3R:1B, 1R:1B, 2R:1G:2B, and 1R:3B) at vegetative stage.

Compound	Molecular formula	Class	RT(min)	Peak area %			
				3R:1B	1R:1B	2R:1G:2B	1R:3B
Endo-Borneol	C_10_H_18_O	Monoterpenoid	13.814	0.47	nd	0.33	nd
Methyleugenol	C_11_H_14_O_2_	Phenylpropanoid	23.786	78.76	79.67	80.77	79.14
Caryophyllene	C_15_H_24_	Squiterpenoid	24.453	13.27	14.53	12.01	13.25
Humulene	C_15_H_24_	Squiterpenoid	25.929	0.52	0.40	nd	1.10
α-Copaene	C_15_H_24_	Squiterpenoid	22.619	1.09	nd	0.95	0.71
α-Cubebene	C_15_H_24_	Squiterpenoid	22.623	nd	0.70	nd	nd
Germacrene D	C_15_H_24_	Squiterpenoid	26.984	2.64	2.14	2.56	2.18
β-Elemene	C_15_H_24_	Squiterpenoid	23.212	nd	0.68	0.61	nd
Caryophyllene oxide	C_15_H_24_O	Squiterpenoid	30.977	1.40	1.89	1.66	2.13
β-Acoradiene	C_15_H_24_	Squiterpenoid	28.046	0.80	nd	nd	nd
Acoradiene	C_15_H_24_	Squiterpenoid	25.941	nd	nd	nd	0.79
Neophytadiene	C_20_H_38_	Diterenoid	40.399	1.04	nd	1.10	0.72
Total phenylpropanoid				78.76077	79.66919	80.76836	79.13866
Total squiterpenoid				19.72658	20.33081	17.7968	20.14198
Total monoterpenoid				0.474692	0	0.334441	0
Total diterpenoid				1.037956	0	1.100399	0.719362
Total				100	100	100	100

“nd” indicates not detected**.** R, G and B represent the red, green and blue llight spectra, respectively, and number before the letters stand for the ratio of each lighrt spectra.

**Table 5:** Chemical profiling of red holy basil under different light spectrum ratios (3R:1B, 1R:1B, 2R:1G:2B, and 1R:3B) at flowering stage.

Compound	Molecular formula	Class	RT(min)	Peak area %			
				3R:1B	1R:1B	2R:1G:2B	1R:3B
Endo-Borneol	C_10_H_18_O	Monoterpenoid	13.814	0.62	0.85	nd	1.21
Methyleugenol	C_11_H_14_O_2_	Phenylpropanoid	23.786	78.12	72.58	77.58	78.29
Caryophyllene	C_15_H_24_	Squiterpenoid	24.453	12.21	14.73	13.93	13.48
Humulene	C_15_H_24_	Squiterpenoid	25.929	0.48	0.57	0.30	nd
α-Guaiene	C_15_H_24_	Squiterpenoid	28.053	1.19	1.40	1.43	1.68
α-Copaene	C_15_H_24_	Squiterpenoid	22.619	1.51	1.93	1.23	0.90
α-Bisabolene	C_15_H_24_	Squiterpenoid	30.983	nd	1.13	nd	nd
Germacrene D	C_15_H_24_	Squiterpenoid	26.984	3.69	4.14	3.63	3.06
Caryophyllene oxide	C_15_H_24_O	Squiterpenoid	30.977	0.94	nd	0.51	0.70
β-Copaene	C_15_H_24_	Squiterpenoid	28.046	nd	1.01	0.64	nd
Neophytadiene	C_20_H_38_	Diterenoid	40.399	0.67	0.95	nd	nd
Phytol	C_20_H_40_O	Diterenoid	49.135	0.57	0.72	0.75	0.67
Total phenylpropanoid				78.11944	72.58	77.57923	78.28789
Total squiterpenoid				20.01809	24.89679	21.6715	19.82613
Total monoterpenoid				0.621864	0.848059	nd	1.212162
Total diterpenoid				1.240606	1.673167	0.749276	0.673827
Total				100	100	100	100

“nd” indicates not detected**.** R, G and B represent the red, green and blue llight spectra, respectively, and number before the letters stand for the ratio of each lighrt spectra.

## Discussion

Our study indicates that variation in light spectrum ratios can have significant impacts in the growth performance of both green and red holy basil cultivars. Measurements of biomass accumulation and plant height in green holy basil indicate that the 3R:1B treatment (high ratio of the red spectrum) had the biggest impact when compared with other light treatments at both the vegetative and flowering stages (Table 1). This was consistent with the reports of Pennisi et al.^30^, who examined R:B LED light effects in sweet basil. Studies in various plant species have shown that the addition of 7.4% green light combined with R and B light results in a greater FW and DW in lettuce^48^. Carvalho et al.^29^ also found that the addition of a small proportion of green light to the 1R:1B ratio increased above ground biomass of sweet basil, when compared with 1R:1B ratio. In this study, the 2R:1G:2B treatment had higher FW at two developmental stages when compared with 3R:1B, 1R:1B and 1R:3B treatments. However, red basil exhibited the opposite effect at the flowering stage, with the 1R:3B treatment eliciting the highest biomass accumulation (FW and DW).

The 3R:1B treatment enhanced photosynthetic ability of green and red holy basil at both developmental stages, while 1R:3B treatment had the lowest photosynthetic performance (Fig. 3A,B). However, increased yield of red holy basil associated with 1R:3B was not related to increase photosynthetic efficiency (Table 1; Fig. 2A). In the present study, the results show higher *gs* under higher ratios of blue light spectra such as 1R:1B, 2R:1G:2B and 3B:1R compared to 3R:1B (Fig. 2C,D), which further correlates with higher *E* values, especially under 1R:3B (Fig. 3G,H). Moreover, the change in *Ci* of green holy basil is likely attributed to *Pn* under various light spectra, as the increase in photosynthetic rate likely results from decreases of *Ci* under high red-light ratio (3R:1B) at the two stages of development (Fig. 3E). Combined R and B light showed an increased *gs* rate compared with R light alone^49^, which decreased guard cell water potential by increasing water uptake and turgor pressure associated with stomatal opening^50^. Moreover, this study showed that the green light spectrum (2R:1G:2B) resulted in a decrease in *E* of green holy basil when compared with 1R:1B. Bouly^51^ reported that green light deactivated blue light’s cryptochrome photoreceptors by eliminating the signal leading to suppressed abscisic acid (ABA) production in guard cells and resulting in decreased stomatal apertures. Photosynthetic activity in mesophyll cells is required for red-light-responsive stomatal opening under fluxes in *Ci*^52^. In this report *Ci* concentration reduced with 3R:1B treatment and was linked with increased *Pn* performance. Under the plant light reaction system, NPQ is a photoprotective process of thermal dissipation of excess excitation energy in PSII, and prevents the accumulation of oxidative damage^53^. The data show that the high blue ratios, (1R:3B) and 2R:1G:2B, reduced *Pn* in red holy basil, while increasing NPQ, when compared with 3R:1B and 1R:1B treatment at flowering stage. These result suggest that higher proportions of blue light might induce light stress with excessive heat during irritation, and lead to reduced photosynthesis in red holy basil, while G light was partly absorbed by chlorophyll and reflected.

In our study, light spectrum treatment affected plant reflectance spectra (an indicator of plant health reflecting chlorophyll and carotenoid content), and water index measure in the plant. Brodersen and Vogelmann^54^ reported that green light contributes more to photosynthesis in deeper leaf layers. Also, they showed that G light in combination with different R and B ratios could influence pigment accumulation by increasing GI and CRI1 at the flowering stage of green holy basil (Fig. 5C,G), potentially leading to increased biomass accumulation (Table 1). The spectral energy transmittance of R and B light corresponds with absorption of light by the pigment^55^. Further, they reported that carotenoid accumulation was higher under high proportions of B light ratios in leafy green plants^56–58^. However, red holy basil maintained high pigment and water content under a high R light ratio (3R:1B). This may explain why the photosynthetic efficiency of red holy basil was enhanced under high R light ratio treatment. The higher photosynthetic performance of red holy basil does not contribute to increased plant biomass under high R light treatment (Table 1) because leaf thickness by high B light treatment will result in a better absorbance and therefore higher plant biomass^59,60^. These results suggest that both stages of green and red holy basil contributed more photosynthetic performance and quantum efficiency of photosystem II at increased B light ratios. However, red holy basil grown under the high B light ratio led to increased plant biomass. Furthermore, G light supplement might have an important role in reducing *E* during the flowering stage of green holy basil.

The light spectrum is one of the key environmental factors affecting metabolite production, and can also modulate secondary metabolite production in plants^61^. Thus, the light spectrum ratio could be related to an enhancement of antioxidant compounds such as phenolics and favonoids. Flavonoids are secondary metabolites including flavonols, flavanones and anthocyanins that accumulate in the stems and leaves of herbaceous plant, and act as antioxidant and photoprotectants in the photosystem^62^. Similarly, the degree of DPPH free radical scavenging is an assay used for antioxidant content evaluation^63^. So far, no effects of light spectrum ratios on antioxidant capacity have been observed in holy basil. Samuoliene^64,65^ reported that R light can improve antioxidant capacity in some lettuce cultivars and sweet basil^30^. Nevertheless, in two lettuce cultivars, flavonoid was enhanced by blue light^66,67^, and a similar experiment by Li and Kubota^25^ showed high anthocyanin content induced by B light in red cross leaves.

In our investigation, high B light spectrum ratio (1R:3B) showed the highest levels of TPC, DPPH scavenging, and anthocyanin content compared to other light spectrum ratios in both green and red holy basils at flowering stage. Moreover, supplementary green light promoted the accumulation of DPPH scavenging in red holy basil, but not in green holy basil. On the other hand, the high ratio of B light spectrum (1R:3B) decreased the accumulation of flavonoid content in both holy basil cultivars (Fig. 6). Experimental studies have shown that the absence of blue light may cause a decrease in flavonoid content in red pak-choi^68^, while no significant flavonoid accumulation was found in sweet basil. This study also found a positive correlation among TPC, DPPH and anthocyanin content at the first principal component from the PCA analyses, while flavonoid content was not correlated (R values greater than 0.25, *P*<0.05) (see Supplementary Document S2; Figure S2). These findings also suggest that a high blue light ratio may play a vital role in stimulating the accumulation of TPC, DPPH and anthocyanin content in both green and red holy basils.

The acclimation of plants in response to environmental conditions may reflect changes in physiological and morphological parameters and in the production of secondary metabolites^69^. The highest production of total dry biomass obtained under higher light radiation indicate that low light intensities may be limiting for the growth of *O. gratissimum* plants^44^. Holy basil has been used as traditional medicinal herbs for a long time^70^, and previous reports have shown that the percentage of secondary metabolite production in *Ocimum* species, varied with different developmental stages^31,44,71^. Moreover, aroma compound composition is fundamental for sensory qualities in herb plant. However, limited studies have reported regarding variation in holy basil in levels of chemical essential oil constituents according to different ratios of light spectrum conditions^31,72^. The main biochemical components of *Ocimum* species are terpenoids and phenylpropanoids including monoterpenes, sesquiterpenes, diterpenes and phenylpropenes. Recently, some studies have reported that the influence of light conditions may lead to aromatic compound modulation^73^. This study demonstrates the action of different light spectrum ratios on metabolism and biochemistry in two cultivars of holy basil at the vegetative and reproductive stages, showing its effects on changing chemical profiles. These results may help to inform lighting increased efficiencies in crop production. The accumulation of monoterpenoids and decreased phenylpropanoids in sweet basil have been shown to be induced under green or yellow light supplement with a background of B and R^29^, while G light has been shown to stimulate the phenylpropanoid pathway in lettuce^74^. Our study showed that total monoterpenoids of green holy basil could not be induced under G light supplement at the vegetative stage while total diterpenoids were stimulated under G light when compared with 1R:1B. However, during flowering stage of green holy basil, the higher levels of phenylpropanoids (methyl eugenol) and diterpenoids were observed under high ratio of B light while total sesquiterpenoids accumulation was associated with high R light proportions. Methyl eugenol is used in many industries from cosmetics, to food, and medical applications. It is used as a flavoring agent in candy and beverages, and as antioxidants and pro-inflammatories^75^. In this study red holy basil at the vegetative stage showed that G added to a background of BR promoted accumulation of total monoterpenoids and diterpenoids, as well as the total phenylpropanoids when compared with 1R:1B and 1R:3B treatment. G light has been described in the past as important for aroma and phenolic compounds accumulation in lettuce^74^ and sweet basil^76^. However, the flowering stage of red holy basil grown under G light supplement inhibited total monoterpenoids accumulation. These result suggest that the production of chemical compounds in both cultivars of holy basil under the developmental stage might be modulated with a change in light spectrum ratios.

The JMP hierarchical clustering analysis revealed that developmental stage and light spectrum ratios were the most important factors on plant biomass accumulation, physiological responses and antioxidant capacities (Fig. 8). In general, development stage appears to be the key determinant whilst lighting ratio effect appears particularly influenced by the level of B light.

## Conclusion

In this study, our results showed that the light spectrum ratios of red (R), green (G) and blue (B) significantly affected on photosynthesis, biomass, antioxidant capacity, as well as secondary metabolite of green and red holy basils cultivated under controlled environments. The photosynthesis was promoted under R:B of 3:1, while, the greatest biomass was found under R:B of 1:3. Adding G to R:B i.e. R:G:B of 2:1:2 could increase the biomass as well as photosynthetic pigment especially in leaf at flowering stage. The R:B of 1:3 significantly increased the DPPH and TPC in leaves of all holy basils at flowering stage, and increased anthocyanin content only in leaves of red holy basils. The total flavonoid content of red holy basil was induced by R:B of 3:1 during both developmental stages. This should be of great value in medicinal plant production, and provide useful information for pharmaceutical and food industries.

## Supplementary Information

Supplementary Information.
