# Supplementary material for: The influence of different light spectra on physiological responses, antioxidant capacity and chemical compositions in two holy basil cultivars
Source: Sci Rep. 2022 Jan 12;12:588. doi: 10.1038/s41598-021-04577-x (PMC8755830; doi:10.1038/s41598-021-04577-x)
Supplement: Supplementary file 1 — Supplementary Information. [file 41598_2021_4577_MOESM1_ESM.pdf]

Supplementary document S1

The influence of different light spectra on physiological responses, antioxidant capacity and chemical compositions in two holy basil cultivars

Panita Chutimanukul[1], Praderm Wanichananan[1], Supattana Janta[1], Theerayut Toojinda[1], Clive Terence Darwell[1], and Kriengkrai Mosaleeyanon[1,*]

***Corresponding author:** Kriengkrai Mosaleeyanon, Tel. +6625646700

Email**:** krieng@biotec.or.th

The relative spectra of the light treatments

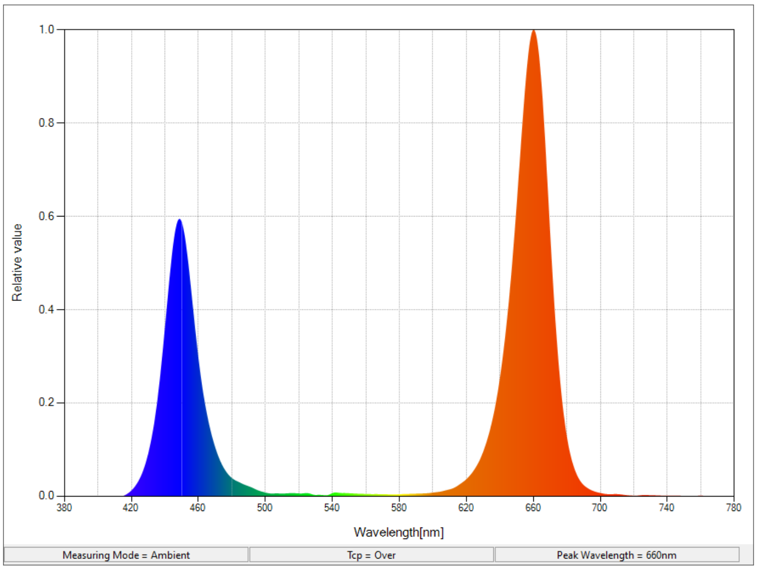

The relative spectra of the light treatment (3Red : 1Blue)

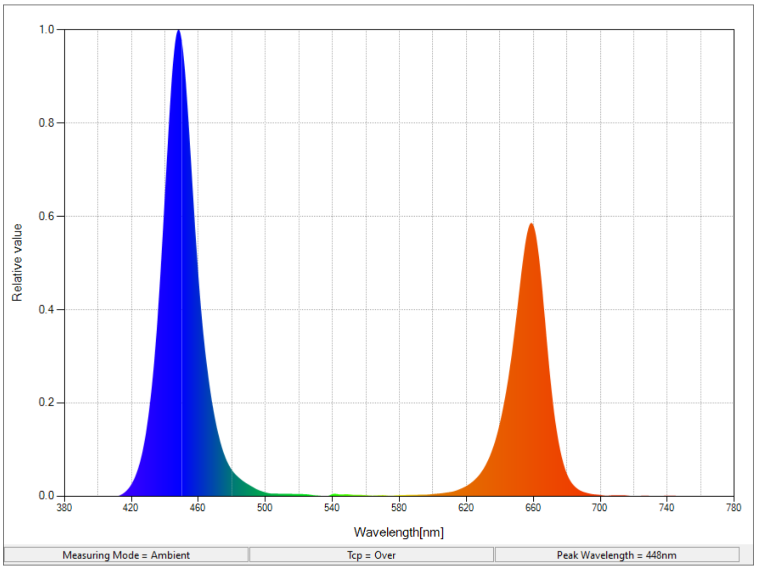

The relative spectra of the light treatment (1Red : 1Blue)

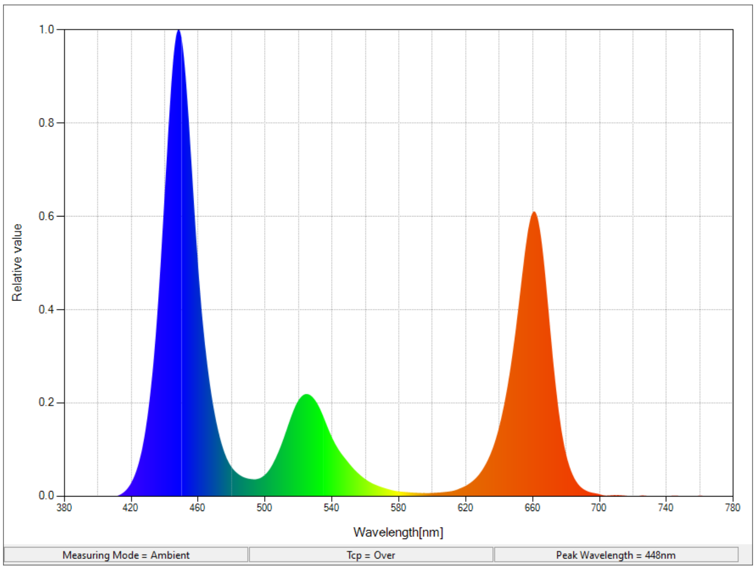

The relative spectra of the light treatment (2Red : 1Green : 2Blue)

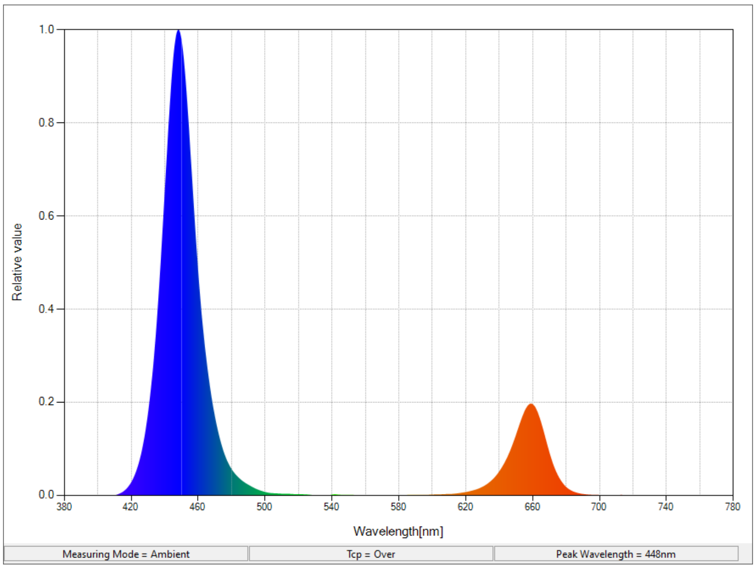

The relative spectra of the light treatment (1Red : 3Blue)

Supplementary document S2

The influence of different light spectra on physiological responses, antioxidant capacity and chemical compositions in two holy basil cultivars

Panita Chutimanukul[1], Praderm Wanichananan[1], Supattana Janta[1], Theerayut Toojinda[1], Clive Terence Darwell[1], and Kriengkrai Mosaleeyanon[1,*]

***Corresponding author:** Kriengkrai Mosaleeyanon, Tel. +6625646700

Email**:** krieng@biotec.or.th

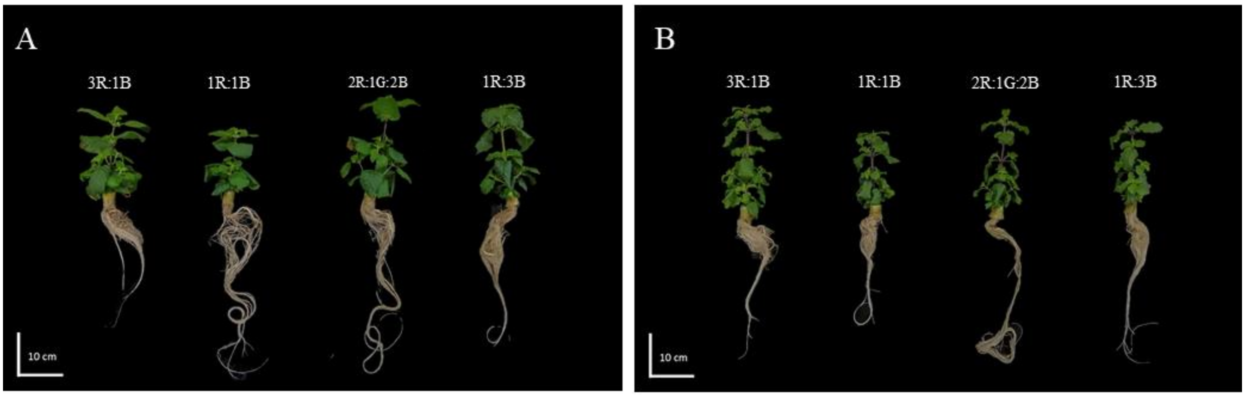

Figure S1. The phenotypes of green holy basil (A) and red holy basil (B) under four light treatments at vegetative stage.

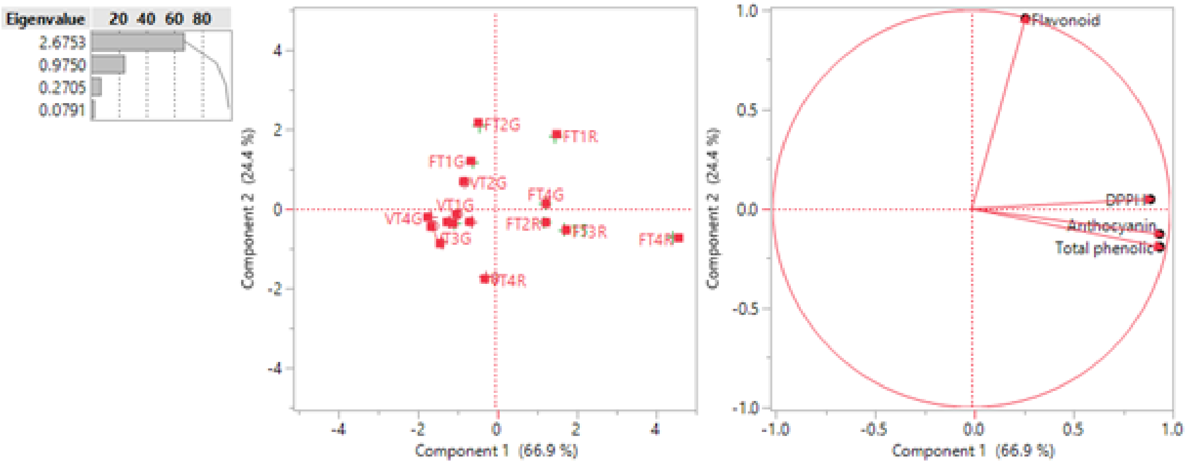

Figure S2. Correlation circle of the principal components analysis (PCA) on the correlation matrix built using data for total phenolic content, flavonoid content, DPPH.

Supplementary Table S1

The influence of different light spectra on physiological responses, antioxidant capacity and chemical compositions in two holy basil cultivars

Panita Chutimanukul[1], Praderm Wanichananan[1], Supattana Janta[1], Theerayut Toojinda[1], Clive Terence Darwell[1], and Kriengkrai Mosaleeyanon[1,*]

Table The details of the daily environment in the plant factory with artificial light

Date	InAirTemp	InAirHumid	InCO2Dens
	C	%	ppm
24/9/2020	23.91	73.88	1009.25
25/9/2020	23.91	73.44	1010.19
26/9/2020	24.17	73.45	1008.39
27/9/2020	24.36	74.16	1018.39
28/9/2020	24.39	73.90	1102.28
29/9/2020	24.40	73.21	1093.60
30/9/2020	24.35	72.35	1056.24
1/10/2020	24.36	73.08	1025.26
2/10/2020	24.12	73.63	1009.24
3/10/2020	24.20	73.90	1032.76
4/10/2020	24.08	74.16	997.59
5/10/2020	24.30	73.78	1019.26
6/10/2020	24.08	73.80	973.79
7/10/2020	24.97	73.85	999.75
8/10/2020	24.21	73.93	1013.45
9/10/2020	24.69	74.13	960.67
10/10/2020	23.82	73.91	1028.79
11/10/2020	24.65	74.02	1015.34
12/10/2020	24.83	73.84	1012.75
13/10/2020	24.55	74.28	1015.54
14/10/2020	23.63	72.14	1014.03
15/10/2020	24.61	70.68	1015.08
16/10/2020	23.39	70.33	1018.92
17/10/2020	24.42	70.44	1018.35
18/10/2020	23.62	70.49	1015.63
19/10/2020	24.60	70.66	1021.03
20/10/2020	23.67	70.87	1024.11
21/10/2020	24.21	70.11	1023.19
22/10/2020	24.15	70.17	1032.00
23/10/2020	24.27	70.48	1026.75
24/10/2020	24.11	70.47	1023.39
25/10/2020	24.18	70.69	1020.15
26/10/2020	22.99	70.65	1096.58
27/10/2020	24.78	71.44	1082.36

28/10/2020	24.38	71.49	1042.61
29/10/2020	24.78	71.48	994.41
30/10/2020	24.51	71.40	1094.83
31/10/2020	24.59	71.58	1018.62
1/11/2020	24.66	70.71	982.59
2/11/2020	24.68	70.10	995.44
3/11/2020	23.58	70.29	1071.12
4/11/2020	24.48	70.80	993.61
5/11/2020	24.58	70.79	1098.98
Average	24.26	72.16	1026.19

I)

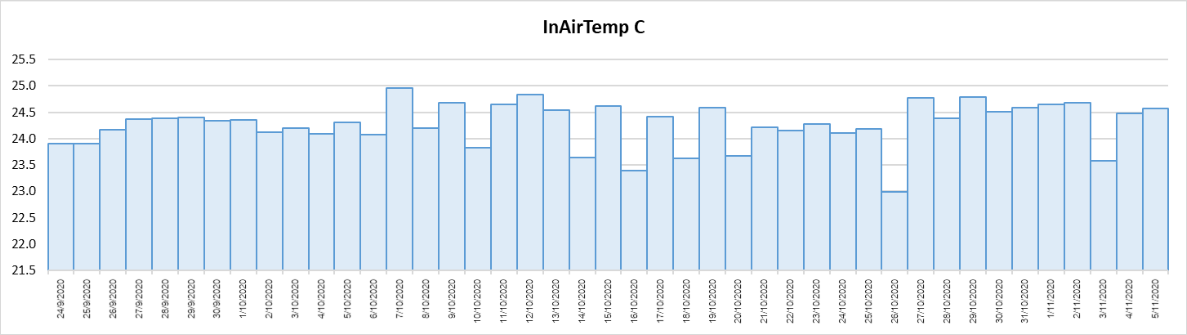

II)

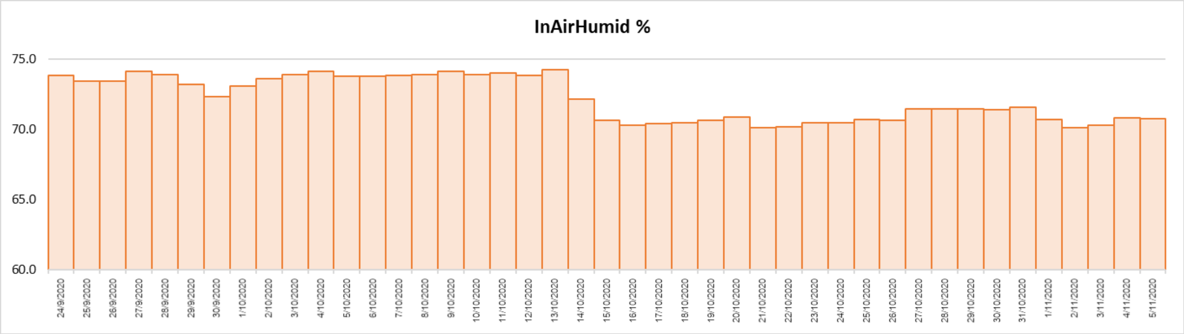

III)

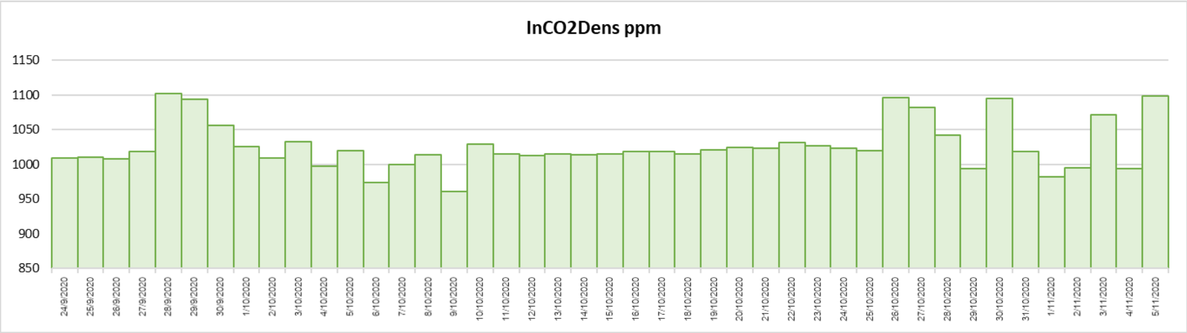

Supplementary Table S2

The influence of different light spectra on physiological responses, antioxidant capacity and chemical compositions in two holy basil cultivars

Panita Chutimanukul[1], Praderm Wanichananan[1], Supattana Janta[1], Theerayut Toojinda[1], Clive Terence Darwell[1], and Kriengkrai Mosaleeyanon[1,*]

*****Corresponding author**:** Kriengkrai Mosaleeyanon, Tel. +6625646700

Email**:** krieng@biotec.or.th

Table Correlation analysis among biomass, physiological parameters and antioxidant capacity of two holy basil cultvars and two developmental stages response to four four different light spectrum ratios.

Attributes	Pn	gs	Ci	E	PhiPS2	ETR	NPQ	NDVI	GI	WI	CRI1	TPC	Flavonoid c	DPPH	Anthocyanin	FW	DW	Height
Pn	1	-0.1048	-0.8357	-0.6236	0.5048	0.3415	-0.1237	0.4525	0.2113	-0.1409	0.2773	-0.3517	0.4898	-0.3294	-0.1947	0.1886	0.1852	0.4006
gs	-0.1048	1	0.0247	0.0994	-0.7776	-0.7901	0.2924	0.1028	-0.6766	0.2553	-0.5608	0.6629	0.3876	0.8569	0.7476	0.4624	0.5308	0.4725
Ci	-0.8357	0.0247	1	0.7843	-0.2301	-0.0663	0.1405	-0.6326	-0.3209	-0.0013	-0.3697	0.251	-0.6056	0.139	0.1767	-0.4438	-0.4684	-0.5668
E	-0.6236	0.0994	0.7843	1	-0.114	0.0347	-0.2308	-0.2939	-0.4404	0.19	-0.3867	0.4274	-0.373	0.1036	0.4466	-0.6293	-0.6118	-0.6759
PhiPS2	0.5048	-0.7776	-0.2301	-0.114	1	0.9756	-0.3183	-0.0884	0.3567	-0.412	0.2776	-0.4696	-0.1974	-0.7844	-0.4513	-0.5515	-0.5858	-0.4116
ETR	0.3415	-0.7901	-0.0663	0.0347	0.9756	1	-0.375	-0.1659	0.2875	-0.4451	0.1853	-0.3494	-0.2868	-0.7504	-0.3829	-0.6568	-0.674	-0.5412
NPQ	-0.1237	0.2924	0.1405	-0.2308	-0.3183	-0.375	1	-0.5972	-0.2767	-0.1102	-0.3502	-0.0829	-0.252	0.2698	0.0491	0.42	0.3286	0.4369
NDVI	0.4525	0.1028	-0.6326	-0.2939	-0.0884	-0.1659	-0.5972	1	0.3844	0.2151	0.5198	-0.0625	0.6736	-0.0584	-0.0321	0.3074	0.3459	0.3199
GI	0.2113	-0.6766	-0.3209	-0.4404	0.3567	0.2875	-0.2767	0.3844	1	-0.1504	0.9545	-0.7141	0.0084	-0.5758	-0.7812	-0.0332	-0.1011	-0.0822
WI	-0.1409	0.2553	-0.0013	0.19	-0.412	-0.4451	-0.1102	0.2151	-0.1504	1	-0.0348	0.029	0.0482	0.088	0.0599	0.184	0.1993	0.0728
CRI1	0.2773	-0.5608	-0.3697	-0.3867	0.2776	0.1853	-0.3502	0.5198	0.9545	-0.0348	1	-0.706	0.1301	-0.5144	-0.7205	0.027	-0.0488	-0.0092
TPC	-0.3517	0.6629	0.251	0.4274	-0.4696	-0.3494	-0.0829	-0.0625	-0.7141	0.029	-0.706	1	0.088	0.7725	0.9166	-0.0884	0.0377	-0.0873
Flavonoid c	0.4898	0.3876	-0.6056	-0.373	-0.1974	-0.2868	-0.252	0.6736	0.0084	0.0482	0.1301	0.088	1	0.2531	0.1502	0.3921	0.5525	0.5054
DPPH	-0.3294	0.8569	0.139	0.1036	-0.7844	-0.7504	0.2698	-0.0584	-0.5758	0.088	-0.5144	0.7725	0.2531	1	0.75	0.3236	0.4047	0.2876
Anthocyanin	-0.1947	0.7476	0.1767	0.4466	-0.4513	-0.3829	0.0491	-0.0321	-0.7812	0.0599	-0.7205	0.9166	0.1502	0.75	1	-0.06	0.0182	-0.0093
FW	0.1886	0.4624	-0.4438	-0.6293	-0.5515	-0.6568	0.42	0.3074	-0.0332	0.184	0.027	-0.0884	0.3921	0.3236	-0.06	1	0.9591	0.9508
DW	0.1852	0.5308	-0.4684	-0.6118	-0.5858	-0.674	0.3286	0.3459	-0.1011	0.1993	-0.0488	0.0377	0.5525	0.4047	0.0182	0.9591	1	0.9342
Height	0.4006	0.4725	-0.5668	-0.6759	-0.4116	-0.5412	0.4369	0.3199	-0.0822	0.0728	-0.0092	-0.0873	0.5054	0.2876	-0.0093	0.9508	0.9342	1
